# Implementation fidelity in a complex intervention promoting psychosocial well-being following stroke: an explanatory sequential mixed methods study

**DOI:** 10.1186/s12874-019-0694-z

**Published:** 2019-03-15

**Authors:** Line Kildal Bragstad, Berit Arnesveen Bronken, Unni Sveen, Ellen Gabrielsen Hjelle, Gabriele Kitzmüller, Randi Martinsen, Kari J. Kvigne, Margrete Mangset, Marit Kirkevold

**Affiliations:** 1 Department of Geriatric Medicine, Oslo University Hospital, Ullevål, P. O Box 4956 Nydalen, 0424 Oslo, Norway; 2 Institute of Health and Society and Research Center for Habilitation and Rehabilitation Services and Models (CHARM), University of Oslo, P.O. Box 1130 Blindern, 0318 Oslo, Norway; 3 Faculty of Social and Health Sciences, Department of Health and Nursing Sciences, Inland Norway University of Applied Sciences, P.O. Box 400, 2418 Elverum, Norway; 4 Faculty of Health Sciences, Oslo Metropolitan University, P.O. Box 4 St. Olavs Plass, 0130 Oslo, Norway; 5 Department of Physical Medicine and Rehabilitation and Department of Geriatric Medicine, Oslo University Hospital, P.O. Box 4956 Nydalen, 0424 Oslo, Norway; 6 Faculty of Health Sciences, Department of Health and Care Sciences, UIT, the Arctic University of Norway, P.O. Box 385, 8505 Narvik, Norway

**Keywords:** Process evaluation, Implementation fidelity, Stroke, Psychosocial intervention, Mixed methods

## Abstract

**Background:**

Evaluation of complex interventions should include a process evaluation to give evaluators, researchers, and policy makers greater confidence in the outcomes reported from RCTs. Implementation fidelity can be part of a process evaluation and refers to the degree to which an intervention is delivered according to protocol. The aim of this implementation fidelity study was to evaluate to what extent a dialogue-based psychosocial intervention was delivered according to protocol. A modified conceptual framework for implementation fidelity was used to guide the analysis.

**Methods:**

This study has an explanatory, sequential two-phase mixed methods design. Quantitative process data were collected longitudinally along with data collection in the RCT. Qualitative process data were collected after the last data collection point of the RCT. Descriptive statistical analyses were conducted to describe the sample, the intervention trajectories, and the adherence measures. A scoring system to clarify quantitative measurement of the levels of implementation was constructed. The qualitative data sources were analyzed separately with a theory-driven content analysis using categories of adherence and potential moderating factors identified in the conceptual framework of implementation fidelity. The quantitative adherence results were extended with the results from the qualitative analysis to assess which potential moderators may have influenced implementation fidelity and in what way.

**Results:**

The results show that the core components of the intervention were delivered although the intervention trajectories were individualized. Based on the composite score of adherence, results show that 80.1% of the interventions in the RCT were implemented with high fidelity. Although it is challenging to assess the importance of each of the moderating factors in relation to the other factors and to their influence on the adherence measures, participant responsiveness, comprehensiveness of policy description, context, and recruitment appeared to be the most prominent moderating factors of implementation fidelity in this study.

**Conclusions:**

This evaluation of implementation fidelity and the discussion of what constitutes high fidelity implementation of this intervention are crucial in understanding the factors influencing the trial outcome. The study also highlights important methodological considerations for researchers planning process evaluations and studies of implementation fidelity.

**Trial registration:**

ClinicalTrials.gov, NCT02338869; registered 10/04/2014.

**Electronic supplementary material:**

The online version of this article (10.1186/s12874-019-0694-z) contains supplementary material, which is available to authorized users.

## Background

In complex interventions in health and rehabilitation, the complexity lies not only within the several interacting components within the intervention itself, but also in the way these components may interact with the context during intervention delivery [1, 2]. A randomized controlled trial (RCT) design is generally the preferred design for evaluating the effect of interventions. However, critics argue that the RCT design oversimplifies cause and effect in complex interventions and that the context of delivering the intervention and the influence exerted by implementers and participants may be ignored in such a study design [1, 3]. Furthermore, there is the risk of evaluating an intervention without knowing if it has been delivered as intended [2]. Thus, successful evaluations of complex interventions should go beyond the traditional outcome evaluation and include a process evaluation [1, 2, 4, 5].

The aim of this implementation fidelity study was to evaluate to what extent a dialogue-based psychosocial intervention was delivered according to protocol [6]. In the following, we will present the concept of process evaluation and the conceptual framework chosen to guide the analysis of implementation fidelity in the present study. Description of the intervention and its theoretical and empirical underpinnings as well as the content and structure of the intervention will be included as background.

### Process evaluation

The concept of process evaluation has become an essential part of designing and testing complex interventions in health and rehabilitation [1, 2]. A clear description of the intervention and its causal assumptions is a prerequisite for conducting a process evaluation and to be able to interpret an intervention’s outcomes. The key questions in process evaluations are concerned with the implementation of the intervention: What was implemented, and how was the intervention implemented? [1]. The concept of implementation can refer to implementation within clinical practice, which may only be endeavored when the intervention has shown effectiveness in outcomes evaluation, or it may refer to the implementation within the context of an effectiveness evaluation (RCT). In this study, we refer to implementation within the context of an RCT which was conducted as part of development and testing of a complex intervention [2].

### Implementation fidelity

The concept of implementation fidelity refers to the degree to which an intervention is consistently delivered according to protocol [1, 2, 5, 7–9]. Implementation fidelity is a potential moderator that may impact the relationship between the intervention and the intended outcome [7]. Assuming there is a well-established theoretical and empirical foundation for the intervention, including identification of active ingredients and their relationship with the intended outcome, evaluating implementation fidelity is essential. When the intervention is consistently delivered according to protocol and a high level of implementation fidelity is achieved, evaluators, researchers, and policy makers can have greater confidence in the research outcomes [1, 7]. Conversely, poor implementation fidelity may help explain non-significant outcomes in intervention studies.

**Fig. 1:**
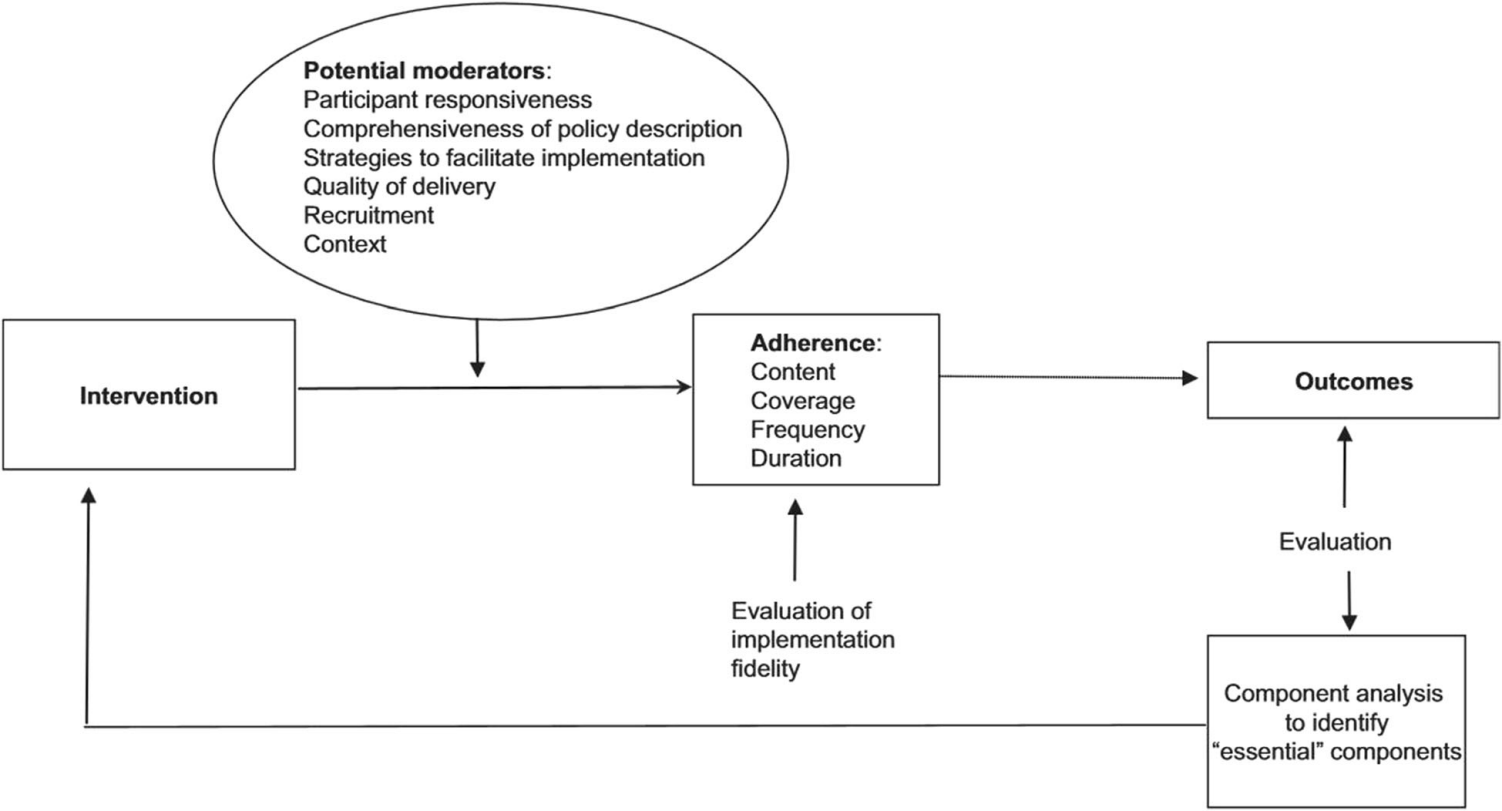
The modified conceptual framework for implementation fidelity [4]

In this study, the conceptual framework for implementation fidelity developed by Carroll et al. [7] and modified by Hasson et al. [4] (Fig. 1) was used to guide our analysis. Carroll et al. [7] argue that an intervention is implemented with high fidelity when there is complete adherence to content, frequency, duration, and coverage. The need for more research to go beyond assessing adherence to make sense of the fidelity concept and to clarify the factors affecting fidelity and their relationship with one another has been emphasized [5, 7]. More attention needs to be paid to the potential moderators of intervention effects and this should be seen in context with the adherence results to form a more complete picture of the overall implementation fidelity.

#### Adherence

The modified conceptual framework for implementation fidelity identifies four elements that constitute adherence (Fig. 1). The content of the intervention refers to the active ingredients that are delivered to recipients. Complete adherence in terms of content allow for flexibility in delivery, as long as the core components are delivered [7]. In addition to content, the assessment of adherence also applies to the coverage, frequency, and duration of the intervention. The concept of coverage, which may be understood as the intervention’s reach, refers to whether or not all the eligible participants were invited to participate, and whether or not the participants who were randomized into the intervention arm of the study received the intervention [4]. Frequency and duration are sometimes referred to as the dose of the intervention, and this refers to whether or not intervention components were implemented as often and as long as planned [4, 7].

#### Potential moderators

As illustrated in Fig. 1 the modified conceptual framework identifies six potential moderators: participant responsiveness, comprehensiveness of policy descriptions, strategies to facilitate implementation, quality of delivery, recruitment, and context. Participant responsiveness refers to how participants are engaged by or respond to the intervention [5] and includes assessments by participants about the outcomes and relevance of an intervention [5, 7]. In this study, participants include both persons receiving the intervention (from now on referred to as participants) and health-care professionals (HCPs) responsible for delivering the intervention (from now referred to as intervention personnel (IP)) [5]. Low participant responsiveness means that the less enthusiastic participants and IP are about an intervention, the less likely the intervention is to be implemented properly and fully [7]. Comprehensiveness of policy description covers intervention complexity and to which degree the intervention is sufficiently and clearly described [5, 7]. Strategies to facilitate implementation often refers to strategies such as provision of manuals, guidelines, training, and supervision through feedback [4]. Facilitation strategies are used to standardize implementation and heighten fidelity. Quality of delivery concerns whether an intervention was delivered in a way appropriate to achieving what was intended. If the content of an intervention is delivered insufficiently, then this may affect the degree to which full implementation is realized. Recruitment as a potential moderator refers to consistency of recruitment procedures, reasons for non-participation among potential participants, and subgroups less likely to participate [5]. The context includes factors that may influence fidelity such as the surrounding social systems, structures and cultures of organization, and concurrent events [5].

### Description of the intervention and its theoretical and empirical underpinnings

#### Theoretical assumptions of the intervention

**Fig. 2:**
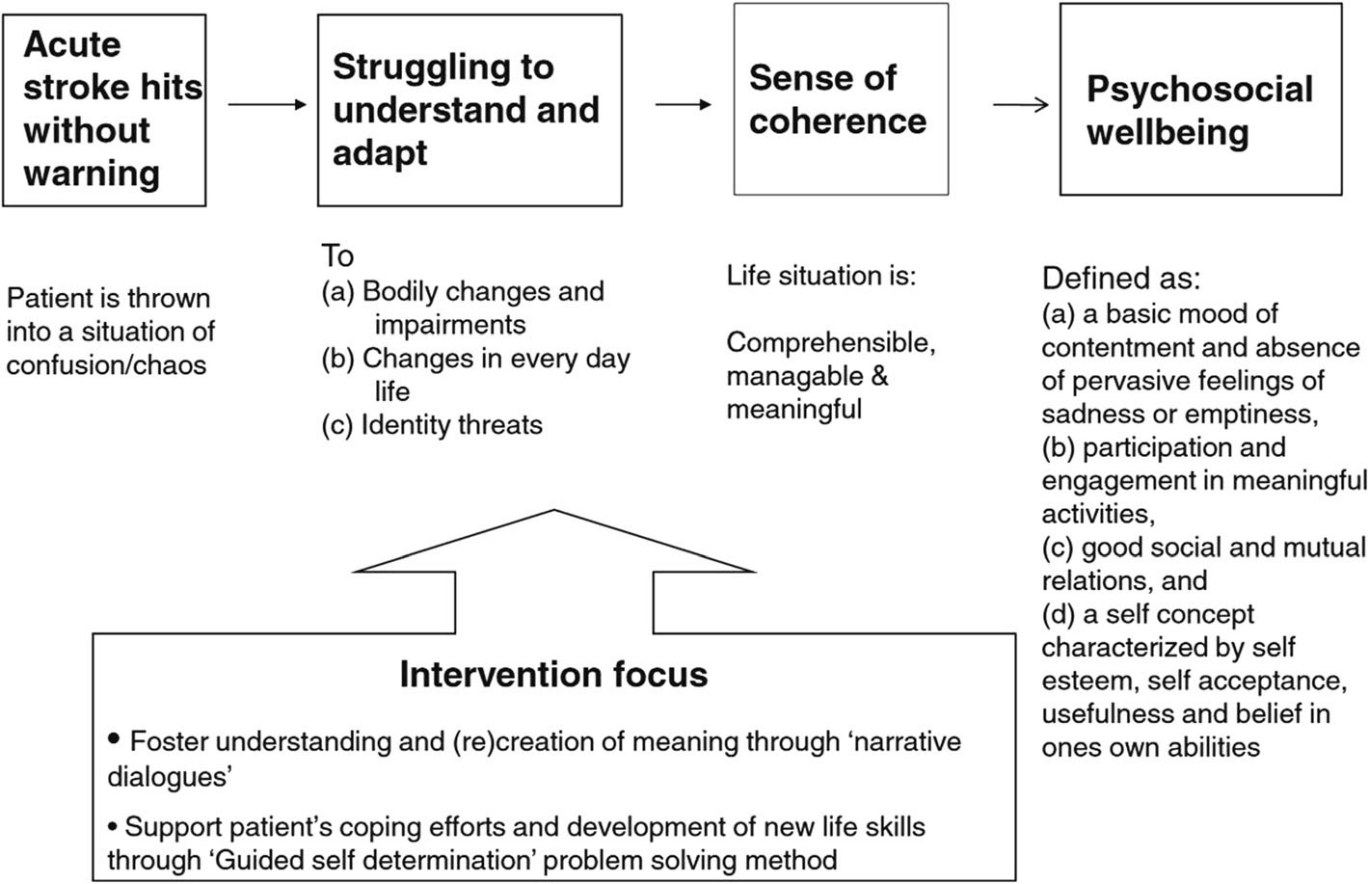
Theoretical structure of the intervention [12]

The intervention evaluated in this study is a dialogue-based individual intervention, tailored for stroke survivors with and without aphasia and designed to be delivered in the early rehabilitation phase starting 4–6 weeks after stroke onset [10]. The overall goal of the intervention is to promote psychosocial well-being following stroke (Fig. 2). Psychosocial well-being was defined as having the following characteristics: (a) a basic mood of contentment and absence of pervasive feelings of sadness or emptiness, (b) participation and engagement in meaningful activities, (c) good social and mutual relations, and (d) a self-concept characterized by self-esteem, self-acceptance, usefulness, and belief in one’s own abilities [11, 12]. Promoting the experience of life events as comprehensible, manageable, and meaningful following Antonovsky’s theory of sense of coherence (SOC) [13, 14], is seen as an intermediate goal for promoting psychosocial well-being [6, 10]. Ideas from the guided self-determination (GSD) method [15] were applied in this intervention to promote coping and development of new life skills. The theoretical underpinnings of the intervention assert that being encouraged and supported to tell one’s story and receiving responses on shared stories from the IP would stimulate reflection and adjustment and strengthen identity, self-understanding, and self-esteem [6, 10]. The intervention was developed and feasibility tested in accordance with the UK MRC guidance for development and evaluation of complex interventions [2]. Development of the intervention, as well as its theoretical and empirical foundation, including its suggested active ingredients, were described in Kirkevold et al. [10] and subsequently evaluated in several feasibility studies [12, 16–19] prior to the evaluation in this RCT study.

#### Structure and content of intervention

**Fig. 3:**
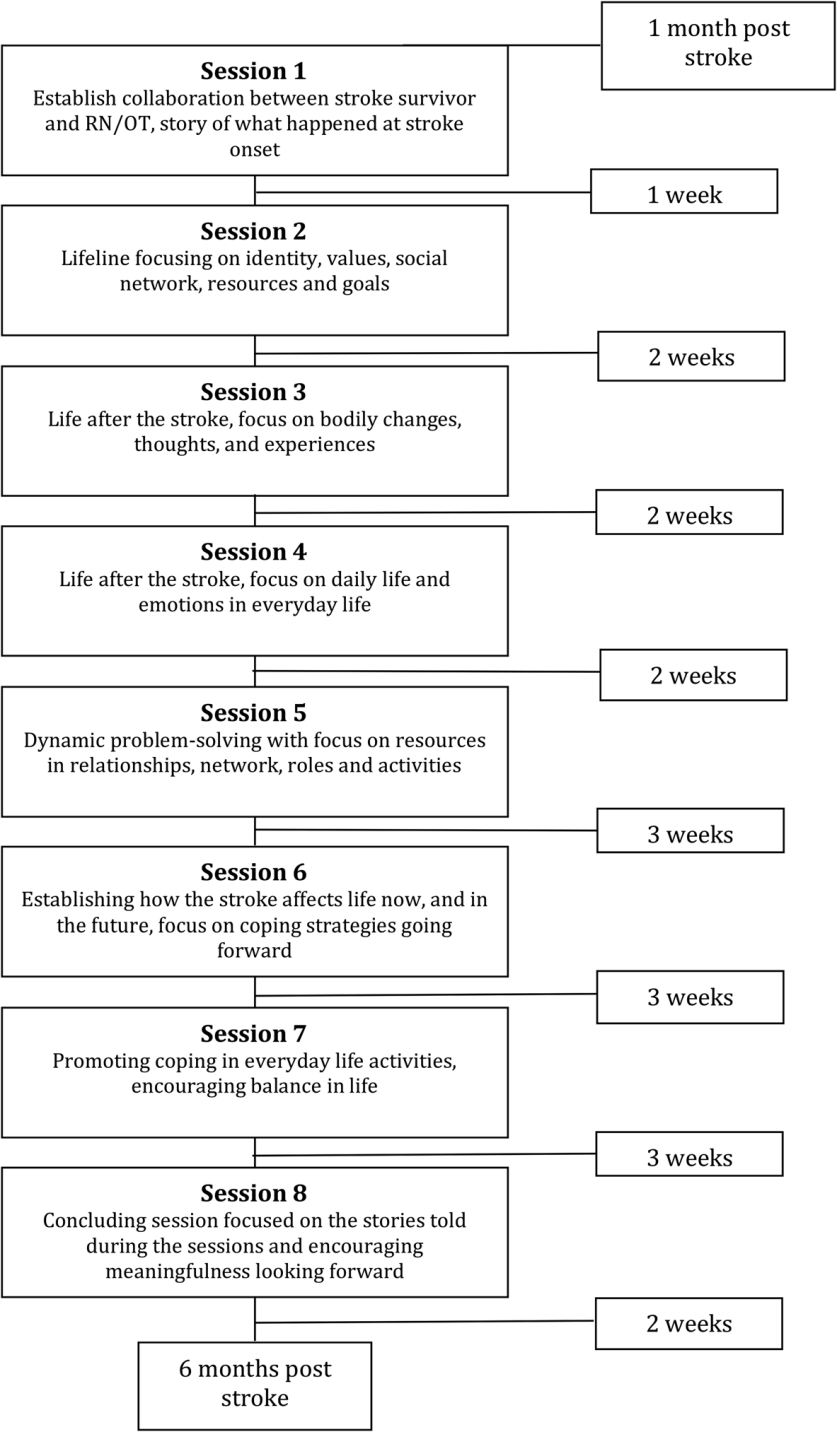
Content and suggested structure of the intervention trajectory [6]

The intervention was designed to be delivered over the course of eight individual sessions between the participant and the IP. The intervention framework outlines a guide of topics to be addressed in each session and a proposed timeline for the intervention trajectory (Fig. 3). In addition to the timeline, a manual describing the goal of each session along with work-sheets addressing each theme was supplied. The IP received training in how to guide each of the sessions and how to use work-sheets as conversation starters. The participant and IP were allowed to individually adapt the order of topics and the time in-between each session to suit the needs of the participant [6].

#### The RCT

**Fig. 4:**
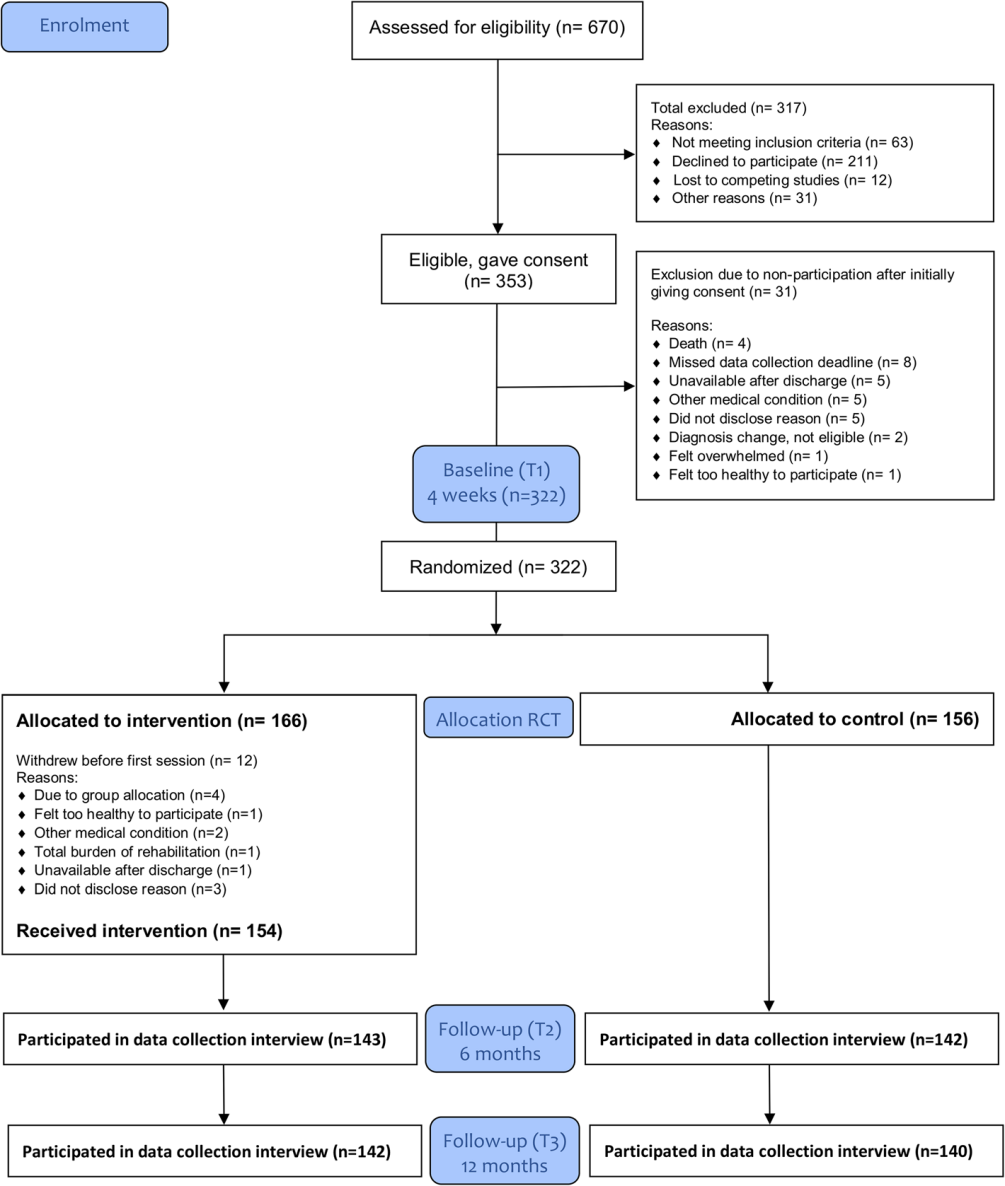
RCT enrolment, group allocation and follow-up at 6 and 12 months

The intervention was tested in an RCT where 11 hospitals in South-Eastern Norway enrolled patients from November 2014 to November 2016 [6]. In total, 322 patients were randomized into the intervention (*n* = 166) and the control arm (*n* = 156) of the trial (Fig. 4). The intervention was delivered in the primary health-care setting mainly in the participant’s home and sometimes at in-patient rehabilitation units if the participant required rehabilitation beyond what the municipality could offer at home. Evaluation of possible effects was assessed relative to baseline scores collected at four to six weeks post-stroke (T1). Structured in-person assessment interviews were conducted by blinded data collectors at six (T2) and twelve (T3) months post-stroke. The General Health Questionnaire-28 (GHQ-28) [20] was the primary outcome measure. Data collection for the RCT was concluded in November 2017. The outcomes of the RCT will be reported in detail elsewhere.

## Methods

### Aim

The aim of this implementation fidelity study was to evaluate to what extent a dialogue-based psychosocial intervention was delivered according to protocol. The research questions in this study were: 1) to what level of fidelity was implementation adherence achieved? and 2) which potential moderating factors affected intervention adherence and overall implementation fidelity?

### Study design and data collection

#### Process evaluation

**Fig. 5:**
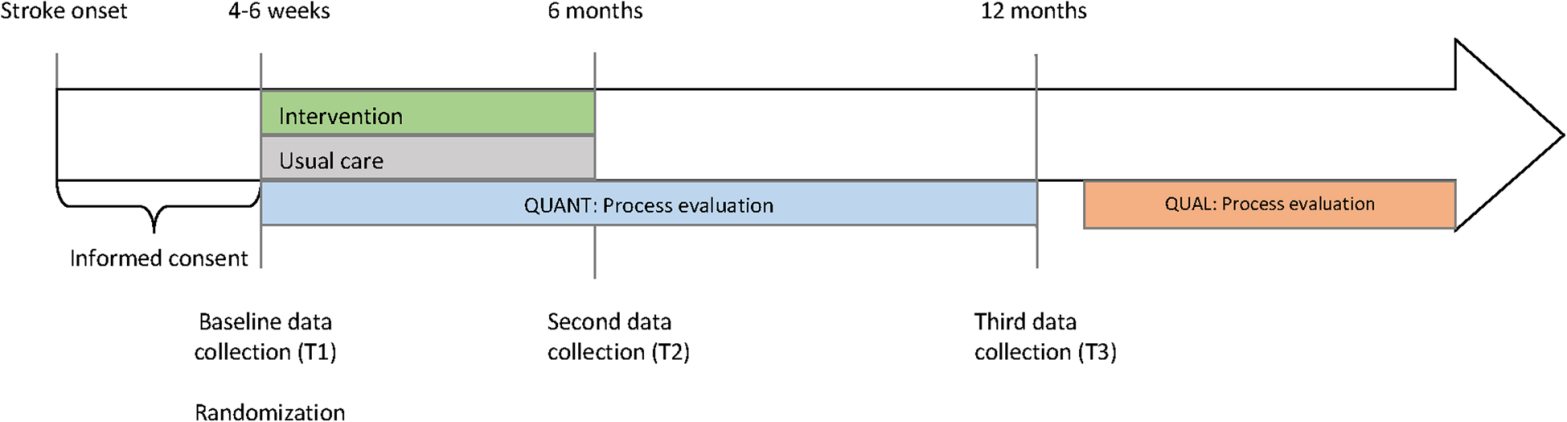
Data collection timeline for RCT and process evaluation

The process evaluation, including the evaluation of implementation fidelity, had an explanatory, sequential, two-phase mixed methods design [21]. Quantitative process data were collected longitudinally along with enrolment, intervention delivery, and outcome data collection in the RCT. To minimize bias in the outcomes of the RCT, the qualitative process data were collected after the last data collection point of the RCT (Fig. 5).

### Participants and data sources

#### Quantitative phase

The sample included in the quantitative phase of the process evaluation consists of key information about the intervention trajectories of all participants (*n* = 166) from the intervention arm of the RCT. The eligibility criteria for the RCT were minimum 18 years of age, medically stable, suffered a stroke within the last 4 weeks, sufficient cognitive function to participate (as assessed by the stroke team) and to give informed consent, and able to understand and speak Norwegian. People with moderate to severe dementia, serious physical or mental illness or severe aphasia were not eligible. The quantitative process data collected in this study consisted of enrolment records from each of the participating centers, intervention records documenting each of the delivered interventions, attrition records from the intervention and control arms of the RCT, and the trial coordinators’ records of data collection time points relative to stroke onset (Table 1). Quantitative process data were collected to document implementation of the intervention within the RCT and to be able to evaluate implementation fidelity.

**Table 1:** Implementation fidelity

	Adherence				Potential moderators					
	Coverage	Content	Frequency	Duration	Participant Responsiveness	Comprehensive-ness of policy description	Strategies to facilitate implementation	Quality of delivery	Recruitment	Context
Quantitative data sources										
Enrolment protocol	X								X	
Intervention records	X	X	X	X						
Attrition records	X			X	X					
Qualitative data sources										
Individual interviews with participants	X	X	X	X	X			X		X
Focus group interviews with intervention personnel		X	X	X	X	X	X	X		X
Trial coordinators’ records							X		X	X

#### Qualitative phase

The sample included in the qualitative phase of the process evaluation consisted of a sample of participants from the intervention arm of the RCT (*n* = 14) and a sample of intervention personnel (*n* = 17). The qualitative process data consisted of individual interviews with participants and focus group interviews with the IP. The qualitative process data were collected to extend the quantitative process data to facilitate an in-depth understanding of the implementation of the intervention and the potential moderating factors affecting implementation fidelity.

##### Individual interviews with participants

A reiterative purposive sampling procedure [22] based on key demographic and stroke-related characteristics were used to recruit participants for the qualitative part of the process evaluation. The sampling criteria were based on the assumption that it would be important to achieve a broad sample with regards to age, gender, caring responsibilities, co-habitation status, type of stroke, aphasia, and stroke severity. Based on earlier research where work-aged stroke survivors with caring responsibilities were identified as particularly vulnerable [18, 19], we wanted to make sure that work-aged stroke survivors with caring responsibilities were represented in the qualitative sample. Thirty-nine participants from the intervention arm of the RCT were invited to participate in individual qualitative interviews. Recruitment procedures were concluded when a sufficient number of participants with different characteristics were included. Eighteen participants consented to participate. Four participants were later excluded; one participant changed his/her mind, one had experienced severe cognitive decline and was not able to answer the questions, one interview was lost due to a tape recorder malfunction, and the last interview was not completed. Consequently, fourteen individual interviews with participants were completed in the period from August 2016 to June 2017. In-depth analysis of the intervention participants’ experiences is reported elsewhere [23].

##### Focus group interviews with intervention personnel

Upon completion of all the intervention trajectories, twenty-one of the IP external to the research team were invited to participate in a focus group interview to share their experiences. IP who were a part of the research team were excluded from the focus group interviews to avoid bias in interpretation of the data. Seventeen of the IP consented to participate. One IP declined participation, one was unable to participate because she had moved to a different geographic region, and the other two were unable to take part because of their workload. Five focus group interviews with IP were conducted from April to July of 2017. A separate qualitative article reporting the results of these focus group interviews and the IP’s experiences with their role will be forthcoming. Table 1 illustrates how the various data sources contribute to different parts of the assessment of implementation fidelity.

### Mixed methods data analyses

The quantitative data set from phase one was analyzed using descriptive statistics, presenting frequencies and mean scores to describe the sample, the intervention trajectories, and the adherence measures. The adherence measurements were assessed against the benchmark content, frequency, and duration outlined in the study protocol [6]. According to the protocol [6], the intervention should consist of 8 individual intervention sessions starting four to eight weeks after stroke onset, following a suggested frequency and total duration of 17 weeks from session 1 to session 8 with completion within 6 months post-stroke (Fig. 3). The intervention trajectory was deemed as complete if ≥6 intervention sessions were completed. As outlined in Additional file 1, a scoring system was devised to clarify measurement of the levels of implementation. The values of each of the variables and a composite adherence score was categorized as; low fidelity, medium fidelity or high fidelity. All statistical analyses in this study were conducted using IBM SPSS Statistics 24 [24].

The interviews with participants and IP were recorded, transcribed, and analyzed separately with a theory-driven content analysis using categories of adherence and potential moderators from the modified conceptual framework of implementation fidelity [5]. The qualitative analysis tool HyperResearch [25] was used to systematize and code the qualitative data sets and to promote an overview of codes across data sets. The moderators are assessed based on the perceived relevance to implementation of the intervention. The results from the qualitative data sets were connected by systematically comparing and contrasting the results from the two samples to examine potential corroborating and contradictory results between the participants and the IP. An excerpt of the qualitative analysis of the potential moderators is outlined in Additional file 2. Subsequently, the results from the qualitative analysis were connected to the quantitative adherence results to assess which potential moderators may have influenced implementation fidelity and in what way.

### Characteristics of participants

#### Participants

The participants in the intervention arm of the RCT were 59% men and 41% women. Their age at admission ranged from 34 to 90 years of age, with a mean age of 66.7 years. In line with the purposive sampling of work-aged participants with caring responsibilities, the process evaluation sample from the intervention arm had a lower mean age (59.6 years) than the age in the total sample. The medical records showed that 85.9% of the intervention group participants in the RCT had suffered an ischemic stroke while 12.8% had suffered a hemorrhagic stroke. A larger proportion of the process evaluation sample had suffered a hemorrhagic stroke (21.4%) than in the total population of the RCT. Additionally, as expected due to the sampling strategy of the qualitative phase, the proportion of participants who were working before the stroke was higher in the process evaluation sample than in the total sample. Please refer to Table 2 for more detailed demographic data and characteristics of the strokes.

**Table 2:** Characteristics of the sample in the intervention arm of the RCT (*n* = 166) and the process evaluation sample of the intervention group (*n* = 14)

	*Total sample from RCT*	*Process evaluation sample*
*Demographic characteristics*	*Mean (S.D)*	*Mean (S.D)*
Age at admission (n = 166;14)	66.7 (12.02)	59.6 (11.9)
	*N (%)*	*N (%)*
Gender (n = 166;14)		
Female	68 (41.0)	6 (42.9)
Male	98 (59.0)	8 (57.1)
Living situation (n = 166;14)		
Live with someone	116 (69.9)	10 (71.4)
Live alone	50 (30.1)	4 (28.6)
Work status pre-stroke (*n* = 163;14)		
Working	61 (37.4)	9 (64.2)
Retired or on disability leave	86 (52.8)	5 (35.7)
Sick leave or under occupational rehabilitation	16 (9.8)	0
Rehabilitation services at baseline (T1) (n = 166;14)		
Physical therapy	98 (59.0)	10 (71.4)
Occupational therapy	73 (44.0)	8 (57.1)
Speech therapy	30 (18.1)	4 (28.6)
Psychologist/psychiatrist	14 (8.4)	3 (21.4)
Home nursing care	56 (33.7)	5 (35.7)
Other rehabilitation services	22 (13.3)	1 (7.1)
No reported rehabilitation services	52 (31.3)	4 (28.6)
Rehabilitation services at 6 months (T2) (*n* = 143;14)		
Physical therapy	51 (35.7)	5 (35.7)
Occupational therapy	9 (6.3)	0 (0.0)
Speech therapy	16 (11.2)	4 (28.6)
Psychologist/psychiatrist	3 (2.1)	1 (7.1)
Home nursing care	17 (11.9)	0 (0.0)
Other rehabilitation services	17 (11.9)	3 (21.4)
No reported rehabilitation services	66 (46.2)	5 (35.7)
*Characteristics of the stroke in acute phase*	*N (%)*	*N (%)*
Type of stroke (*n* = 149;14)		
Ischemic	128 (85.9)	9 (64.3)
Hemorrhagic	19 (12.8)	3 (21.4)
Not specified	2 (1.3)	2 (14.3)
Stroke symptom localization (*n* = 144;13)		
Right side	65 (45.1)	6 (46.2)
Left side	70 (48.6)	6 (46.2)
Bilateral	7 (4.9)	1 (7.7)
Not relevant	2 (1.4)	0 (0.0)
Language impairment at assessment in hospital (*n* = 129;13)		
Yes	44 (34.1)	7 (53.8)
No	85 (65.9)	6 (46.2)
NIHSS classification (*n* = 126;14)		
0–5	85 (67.5)	9 (69.2)
6–10	28 (22.2)	3 (23.1)
11–15	13 (10.3)	1 (7.7)
16+	0 (0.0)	0 (0.0)

#### Intervention personnel

Including the members of the research team, a total of twenty-seven IP, 20 registered nurses (RNs) and seven occupational therapists (OTs), were certified to deliver the intervention in this study (Table 3). In the process evaluation sample, the proportion of RNs and OTs were approximately the same as in the total sample. On average, the IP in the total sample had 17.8 years of clinical experience and, on average, 9 years clinical experience working with persons with stroke. In the process evaluation sample, the clinical experience in general and with stroke in particular was slightly higher than in the total sample. At the time of the study, 51.9% of the IP in the total sample and 76.5% of the process evaluation sample were employed in a clinical position working directly with patients. Please refer to Table 3 for more details about the characteristics of the IP.

**Table 3:** Characteristics of intervention personnel in the RCT (*n* = 27) and in the process evaluation sample (n = 17)

	*Total sample*	*Process evaluation sample*
*Demographic characteristics*	*N (%)*	*N (%)*
Professional background (*n* = 27;17)		
Nurse	20 (74.1)	13 (76.5)
Occupational therapist	7 (25.9)	4 (23.5)
Highest Educational level (n = 27;17)		
Bachelor degree	4 (14.8)	3 (17.6)
Continuing education	9 (33.3)	9 (52.9)
Master’s degree	8 (29.6)	5 (29.4)
PhD	6 (22.2)	0 (0.0)
Primary employment (n = 27;17)		
Clinical practice	14 (51.9)	13 (76.5)
Education and research	13 (48.1)	4 (23.5)
Type of employment in clinical practice (n = 14;12)		
Stroke unit or specialized rehabilitation unit	10 (71.4)	9 (75.0)
General practice	4 (28.5)	3 (25.0)
Part of the research team (n = 27;17)		
Yes	6 (22.2)	0 (0.0)
No	21 (77.8)	17 (100.0)
*Clinical experience*		
Clinical experience, in years (*n* = 26;17)		
Mean	17.8	18.9
S.D	10.06	9.17
Min-Max	2–40	7.5–40
Clinical experience with stroke survivors, in years (n = 26; 17)		
Mean	9.0	9.8
S.D	6.05	5.25
Min-Max	1–20	2–20

## Results

The presentation of results is structured according to the conceptual framework for implementation fidelity (Fig. 1). Initially, the quantitative measures of adherence and the calculated levels of intervention fidelity based on the quantitative analyses are presented separately. Subsequently, the section is structured according to the adherence measures moderated by the most prominent potential moderating factors, and presented in combination based on the mixed methods analysis.

### Adherence and level of intervention fidelity

#### Adherence

The intervention records show that the participants completed, on average, 7.4 sessions (SD: 1.5, 95% CI: 7.2–7.6) of the maximum 8 sessions. In total, 140 participants (90.9%) received a complete intervention program of ≥6 sessions (Table 4).

**Table 4:** Number of sessions delivered

Number of sessions delivered (*n* = 154)	N (%*)
**8**	**120 (77.9)**
**7**	**15 (87.7)**
**6**	**5 (90.9)**
5	4 (93.5)
4	2 (94.8)
3	3 (96.8)
2	3 (98.7)
1	2 (100)
Total number	154 (100/100)

*Cumulative percentage

The concepts of frequency and duration is represented by three variables: two variables for timeliness and one for duration. Analyses of the detailed intervention records show that the first intervention session was completed, on average, 49 days post-stroke (timeliness of start), while the protocol estimated the start of the intervention to be within 60 days of stroke onset. Furthermore, the last session of the intervention was completed, on average, 12.6 days before the exact 6-month post-stroke date (timeliness of end). This indicates that overall the intervention trajectories from start to finish were within the limits set in the protocol. The duration of the interventions ranged from a minimum of 1 week to a maximum of 27 weeks, being, on average, 16.88 weeks (Table 5). A closer look at the results demonstrates variance in the adherence measures due to outliers that distort the central tendencies and veils the more nuanced picture. This underscores the need to look closer at these adherence variables and the levels of fidelity.

**Table 5:** Frequency and duration in complete vs. incomplete intervention trajectories

Frequency and duration	All interventions (1–8 sessions)	Incomplete interventions (< 6 sessions)	Complete interventions (≥6 *sessions*)
*Timeliness – start (days) (n = 147;11;136)*			
Mean (s.e)	49.1 (1.13)	50.18 (4.98)	49.01 (1.16)
95% CI	46.87;51.33	39.08;61.29	46.73;51.30
SD	13.66	16.53	13.47
*Timeliness – end (days) (n = 150;10;140)*			
Mean (s.e)	−12.57 (2.47)	−87.10 (11.24)	−7.24 (1.83)
95% CI	−17.44;−7.69	−112.52;−61.68	−10.86;−3.62
SD	30.22	35.54	21.65
*Duration (weeks) (n = 146;10;136)*			
Mean (s.e)	16.88 (0.35)	6.3 (1.7)	17.65 (0.25)
95% CI	16.18;17.58	2.45;10.15	17.15;18.16
SD	4.24	5.38	2.96

We isolated 90.9% of the intervention trajectories where a complete intervention (≥6 *sessions*) was delivered and compared those with the 9.1% who received an incomplete intervention trajectory. This comparison illustrates how the incomplete interventions with irregular timeliness data influenced the timeliness of end and duration in particular (Table 5).

#### Levels of fidelity

Based on the scoring system devised for the levels of fidelity (Additional file 1), the values of the adherence variables were placed in three categories: low fidelity, medium fidelity and high fidelity (Table 6).

**Table 6:** Clarification of adherence measures and composite score

Adherence measures	Low fidelity N (%)	Medium fidelity N (%)	High fidelity N (%)
*Content and coverage*			
Number of sessions (n = 154)	8 (5.2)	6 (3.9)	140 (90.9)
*Frequency and duration*			
Timeliness – start (n = 147)	5 (3.4)	23 (15.6)	119 (81.0)
Timeliness – end (n = 150)	28 (18.7)	45 (30.0)	77 (51.3)
Duration (*n* = 146)	8 (5.5)	17 (11.6)	121 (82.9)
Composite score			
Adherence (n = 146)	6 (4.1)	23 (15.8)	**117 (80.1)**

Based on the number of delivered sessions alone, 90.9% of the interventions were implemented with high fidelity. The most challenging part of implementation adherence was completing the intervention sessions in time for the second data collection point at 6 months post stroke. Only 51.3% of the interventions satisfied the criteria for high fidelity in terms of timely completion of the intervention. In all the other adherence measures (number of sessions, timeliness of start, and duration of intervention), from 80 to 90% of the interventions satisfied the criteria of high fidelity. Based on the composite score, 80.1% of the interventions were implemented with high fidelity (Table 6). These levels of fidelity are based entirely on the quantitative material and should be interpreted with caution. In the following, potential moderating factors will be considered to give a broader view of their impact on adherence and overall implementation fidelity.

### Content moderated by participant responsiveness, comprehensiveness of policy description, strategies to facilitate implementation, and quality of delivery

#### Participant responsiveness

The qualitative interviews with participants and IP showed that individual adjustments were made in terms of the content, frequency, and order of the themes/sessions described in the intervention manual (Fig. 3). In general, the participants found the session themes highly relevant as foundations for reflection and as guidance during the intervention.

The IP reported that the thematic work-sheets were useful conversation starters, while the participants were more divided in their opinions of the work-sheets. Some participants enjoyed working actively on the work-sheets as they felt that these helped them to reflect on their values, aims, and future life. Others were reluctant to use the work-sheets for taking notes as that was perceived as a form of homework. Some perceived the work-sheets to be less relevant to their particular adjustment process and preferred not to use them. The interaction with the themes raised and work-sheets used during the intervention trajectory is an important factor in assessing participant responsiveness, including both the participants’ and IP’s responsiveness to the themes and work-sheets.

The narrative approach applied in the intervention was identified by the participants as an important part of the intervention, even though they used layman’s terms to describe this. It appeared to be especially important for the participants to be able to tell their stories to an attentive listener and to take part in a dialogue where they could voice their concerns and receive validation – this was the most useful element according to the participants. Being able to troubleshoot perceived adjustment problems with the IP rather than the IP supplying the answer to their problem was also highlighted as particularly helpful.

The way both the participants and the IP interacted with the intervention may have mediated the content delivered during the sessions. As the RCT study was not conducted as part of an established health care service, all the IP were voluntary participants who delivered the intervention as an addition to their ordinary work. This indicated that they were highly motivated and committed to participate, which can be perceived as a mediator for conducting the intervention in line with the protocol. Both the participants and the IP emphasized the importance of the close working relationship they developed throughout the intervention trajectory. However, the IPs reported that some of the participants struggled to distinguish between ordinary health-care services and the sessions that were part of the intervention. This lack in distinction may be an indication of low participant responsiveness. The quality of the interaction between the participant and the IP was perceived as an important mediator to implementation fidelity. The IP who succeeded in conveying the core components of the intervention appear to have promoted positive responses to the intervention content in the participants, which, in turn, may have facilitated the content delivery and encouraged implementation fidelity.

#### Comprehensiveness of policy description

The IP were certified through a 3-day training program consisting of lectures, practical training exercises, group reflection and discussions, and individual reading of specific literature. The content of the training program is reported in detail in Kirkevold et al. [6]. A detailed manual describing the content and a suggested structure of each of the 8 sessions in the intervention was thoroughly presented and distributed to the IP during the training program [6]. In the focus group interviews, the IP described the manual as a useful tool that they had used systematically in their intervention delivery. Specifically, it was seen as helpful to have the manual to study the intention and conceptual framework of the intervention before each session. Some of the IP experienced that the time between the training program and their first intervention was too long. This prolonged time without practical experience with delivering the content of the intervention was perceived to be a negative factor that may have hampered the implementation fidelity.

#### The strategies to facilitate implementation

From a research perspective, strategies to facilitate implementation appears to be essential in the evaluation of implementation fidelity. Strategies to facilitate implementation were planned and applied during the course of the implementation of the intervention. The IP were supplied with a detailed manual as described over, and the 3-day training program was mandatory for all IP. In addition, the IP were offered supervision individually and in groups during the intervention delivery to facilitate uniform delivery. The written procedures, training program, and supervision during the study period were evaluated as informative and adequate. However, the IP did not emphasize the aspect of strategies to facilitate implementation as clearly as the other moderators. The reason for this may be that the IP outside the research team were more focused on the substantial contribution of the intervention in the focus group interviews, and consequently, not as sensitive to the research aspects of the trial.

#### Quality of delivery

Six of the 27 individuals delivering interventions were part of the research team (Table 7). These six individuals delivered the intervention to 51.3% of the participants, while the 21 externally engaged IP delivered the intervention to the remaining 48.7%. The six individuals from the research team each delivered the intervention to three up to 33 participants, with 13.2 each on average. While the 21 remaining IP each delivered the intervention to one up to nine participants, with 3.6 each on average (Table 7).

**Table 7:** Characteristics of delivered interventions

*Characteristics of the delivered interventions*	External intervention personnel (*n* = 21)	Members of research team (*n* = 6)	Total
Number of interventions delivered (n = 154)			
Total N (%)	75 (48.7)	79 (51.3)	154 (100)
Mean (S.D)	3.6 (2.27)	13.2 (10.94)	5.7 (6.60)
Min-Max	1–9	3–33	1–33

The variation in number of intervention trajectories that each of the external IP delivered deviated from the protocol which suggested that each IP should deliver the intervention to at least four participants. The focus group interviews with the IP revealed that they felt a bit inept during their first intervention sessions, but gradually became more comfortable, flexible, and able to adjust the intervention to the complexity of the individuals’ needs after conducting several interventions. This suggests that increased experience would be conducive to increased individualization and quality of delivery.

### Coverage moderated by recruitment and context

#### Recruitment

The enrolment period for the RCT spanned from November 2014 to November 2016. The trial coordinators’ protocols and the enrolment protocol show that, of the patients assessed for eligibility, approximately 50% consented to participate (Fig. 4). The enrolment record shows that several of the participating centers had unplanned cessations in their recruitment efforts and the recruiters documented that the consent process was not always followed up due to a number of practical and logistical reasons in the hospitals. Additionally, other rival studies targeting the same population were a concern in a few of the hospitals. Unfortunately, due to unexpected periods of enrolment cessation and rival studies we have to assume that the enrolment record underestimates the number of patients who should have been assessed for eligibility. Lower coverage, in terms of not all eligible participants being screened for participation due to recruitment issues, may have influenced the sample recruited for the study. Unfortunately, we do not know if those patients who were not screened for eligibility were significantly different from those who were screened, as the Medical Ethics Committee does not allow collection of data about persons who have not consented to participate in the study.

#### Context

The second aspect of coverage is concerned with whether or not the participants randomized to the intervention arm received the intervention. The intervention records show that in 154 of the 166 (92.8%) intervention trajectories one or more intervention sessions were delivered. Twelve participants chose, for various reasons, not to participate in any intervention sessions (Fig. 4). The intervention records and the attrition record show that none of the participants who completed the intervention trajectory (≥6 sessions) were lost to follow-up at 6 months post-stroke. In the intervention arm of the study, there was an attrition rate of 13.9% from baseline (1 month) to the 6-month data collection point (23 out of 166 participants). As a comparison, at 6 months post-stroke, the attrition rate from the control arm of the intervention was 9% (14 out of 156) (Fig. 4). The intervention participants had busy schedules with concurrent rehabilitation efforts, doctor’s appointments, returning to work, and caring for underage children or other family members to mention a few contextual issues that sometimes came in conflict with participation in the intervention. Seen in context with the expected compliance with the intervention, these issues may in part explain the higher attrition rate in the intervention arm of the study compared to the control arm. Other reasons for drop-out were connected to participants’ health condition, either feeling too healthy or too sick to participate.

### Frequency and duration (dose) of intervention moderated by participant responsiveness and context

#### Participant responsiveness

The concepts of frequency and duration is represented by three variables in our analysis: two variables for timeliness and one for duration (Table 4 and Table 5). The data demonstrates variance in the adherence measures which points to challenges in intervention delivery according to protocol. The focus group interviews with the IP indicated that variability in frequency and duration may be linked to the participants’ expected effect of the intervention and expected suitability of this kind of intervention in each individual case. Participants who completed the intervention but at the same time perceived themselves to be too healthy to participate, expressed a need for fewer sessions, and this impression was supported by the experiences of the IP. Conversely, some participants who initially thought they did not need this intervention discovered that it was a useful contribution in their recovery and rehabilitation trajectory. And thus, they welcomed the idea of a longer intervention trajectory with a greater number of sessions or less frequent sessions in the beginning to stretch the trajectory out.

#### Context

The interviews with the IP and the trial coordinators’ records indicated that in some cases it was difficult to adhere to the frequency and duration guidelines in the protocol because of logistical and contextual issues. The participants’ concurrent use of regular rehabilitation services in the municipality and in secondary health care institutions was pervasive (Table 2). The rehabilitation service use declined from one to six months post stroke, but a large portion of the participants were receiving regular rehabilitation services throughout the intervention trajectory. The interviews with the IP and trial coordinators’ records in some cases highlighted challenges in scheduling intervention sessions due to the participants’ busy schedules. The intervention sessions often had to be scheduled around public holidays, the participants’ travels and participants’ scheduled rehabilitation both in-patient and out-patient rehabilitation in the municipality. In some cases, the IP’s schedule, vacation and sick-leave also came in conflict with the proposed frequency and duration. These contextual factors may have made the implementation of the intervention more challenging.

## Discussion

The aim of this implementation fidelity study was to evaluate to what extent a dialogue-based psychosocial intervention was delivered according to protocol, specifically exploring the level of fidelity with which the implementation adherence was achieved and which potential moderating factors affected intervention adherence and overall implementation fidelity. The study also highlights important methodological considerations for researchers planning process evaluations and studies of implementation fidelity.

Based on the composite implementation fidelity measures, we conclude that four out of five interventions were implemented with high fidelity. There were several factors which contributed to moderation of adherence to the protocol and to implementation fidelity overall. Most importantly and in order of importance, participant responsiveness, context, quality of delivery, comprehensiveness of policy description, and recruitment were the most prominent moderating factors of implementation fidelity.

### Participant responsiveness

This study shows that the participants who were committed to participation did so because they felt the intervention was a positive influence in their adjustment process – their responsiveness to the intervention was favorable. However, some of the patients participated for other reasons than expressed needs for support or usefulness for their own psychosocial well-being. Personal characteristics, such as high moral standards of finishing something you have consented and committed to, as well as the quality of the relationship between the participant and the IP, may explain why some of the participants completed 8 sessions despite expressing a lack of usefulness of the sessions in their own particular recovery and rehabilitation. These results underscore the need to assess which patients would possibly benefit from this intervention to be able to target this intervention to the correct demographic. It is difficult to identify which participants will most likely benefit from the intervention without also including the outcomes of the RCT. In the RCT, specific characteristics of participants who may or may not benefit from the intervention will be identified. The conclusions regarding future eligibility will therefore have to be made based on the results of this study combined with the forthcoming results of the RCT.

IP with a greater number of interventions and higher participant responsiveness had better integration of the core components of the intervention and greater confidence in their mastery of delivering the intervention. Presumably, the IP who led several interventions and those who were not substantially delayed in delivering their first intervention, relative to their certification, may have had better chances of sufficiently integrating the intervention’s core components and methods. This also means that the IP from the research team who had developed the intervention and who presumably had the core components of the intervention highly integrated in their practice, in addition to having completed a larger proportion of the interventions, had a substantial advantage in conducting interventions with high fidelity. The IP’s responsiveness to the intervention and the extent of their practical experience with the intervention and knowledge of the intervention manual were important contributions toward implementation fidelity in this study. The reliance on IP with high motivation, commitment, and responsiveness has implications for potential future implementation in regular clinical practice. The IP recruited in this study had a special interest in stroke rehabilitation and all had higher formal education than what is expected in an average municipal home health care setting. This will have implications for training of IP and strategies to facilitate implementation in line with the protocol if the intervention is to be implemented in regular clinical practice. The IP should possibly be chosen based on expressed motivation and commitment to the intervention.

### Context

In this study, the concurrent use of regular rehabilitation services in the municipality and in secondary health care institutions as well as logistical issues concerned with frequency and duration were important parts of the context that moderated implementation fidelity. The rehabilitation service use declined from T1 to T2, but a large portion of the participants were still receiving regular rehabilitation services at six months post-stroke (Table 2). The pervasive use of rehabilitation services along with the intervention will make it more difficult to distinguish the effect of the intervention alone. The logistical issues concerned with scheduling intervention sessions were mostly related to the duration and timely ending of the trajectory. However, the conditions under which the RCT was conducted is probably a good replication of what one would encounter in the natural clinical setting. Establishing good eligibility criteria and strategies for implementation would, thus, be important before implementation into regular clinical practice.

### Quality of delivery and comprehensiveness of policy description

Quality of delivery in this study was closely related to comprehensiveness of policy description, participant responsiveness, and the number of intervention trajectories conducted by each IP. The description of the intervention and the training program the IP had to complete before certification were important prerequisites for quality of delivery. One challenge to overcome in implementation in regular clinical practice would possibly be to ensure sufficient time for training and supervision.

### Recruitment

Recruitment was an important moderator to the adherence measurement of coverage which covers whether or not all eligible participants were asked to participate and whether or not participants who were randomized into the intervention arm actually received the intervention. The recruitment of participants to this study was not optimal, only 50% of the eligible participants consented to participate, and there were contextual issues that hampered recruitment. The implementation fidelity might have been higher if the eligibility criteria had not been as broad as they were. If the feasibility studies had given clear guidance in terms of who might benefit from this intervention, the eligibility criteria might have been more targeted and we might have avoided drop-outs due to the participants’ self-perceived ineligibility. Nonetheless, the overall coverage in the second sense was high: only 9.1% of the participants who started the intervention received less than a complete intervention (< 6 sessions). In a comparable study [26] effectiveness of the intervention was demonstrated despite the fact that 22.3% of the participants received less than a complete intervention. Another promising result is that none of the 90% of participants who completed the intervention (≥6 sessions) were lost to follow up at six months.

The results of this study strongly indicate that the intervention was delivered according to protocol. This study of implementation fidelity has shed light on which aspects of implementation were successful and which aspects that were more challenging in terms of fidelity. The results provide important insight in the implementation of this intervention which will be of great importance in the interpretation of the forthcoming RCT outcomes.

### Methodological discussion

The methodological strengths and limitations of this implementation fidelity study are largely connected to the design of the process evaluation, the types of data available for analysis, the quality of the data, and the interpretations made based on the analyses. There were no easily available specific models for process evaluation for this particular intervention, which meant that the research team had to design a plan for the process evaluation, what kind of data to collect, in what way, and how the data should be analyzed [6]. In this section, we will discuss some methodological considerations we believe may have relevance beyond the evaluation of this particular intervention.

The sampling plan for data sources and participants in the process evaluation is an important aspect in securing high quality data in the evaluation. In this study, the sampling plan was decided when the process evaluation was designed. Judgements were made in terms of which data sources were readily available through the process data of the RCT itself, and which data sources would have to be added for the purpose of the process evaluation. The sampling for the qualitative phase of the process evaluation was a particular challenge. Due to strict research ethics guidelines, the opt-in approach where participants are informed about the study and then actively communicate their consent to participate, had to be used in the sampling of participants. This approach is known to require a higher number of invitations to reach an acceptable sample size and is also known to introduce selection bias [27, 28]. A common opt-in selection bias is that those who opt-in may be experiencing less problems or challenges connected to their diagnosis and are less disadvantaged than those who chose not to opt-in [28]. One way to ameliorate this selection bias is to specifically target those with the specific characteristics you want to study by applying a purposive sampling strategy such as the one applied in this study. The data available on the non-responders is not sufficient to compare the two groups. Thus, we cannot be sure that the sample recruited in the qualitative phase of the process evaluation was unbiased.

From a research perspective, strategies to facilitate implementation appears to be important, but the IP did not emphasize this aspect as clearly as the other moderators. The lack of emphasis was probably due to the exclusion of the IP from the research team in the focus group interviews, which was decided to avoid bias. In avoiding one kind of bias, we may have inadvertently introduced a different bias, which may need to be addressed in future evaluations of implementation fidelity.

The individual adaptations, which were encouraged in this intervention [6], and which are the rule rather than the exception in complex interventions [2], complicate the measurement of adherence. The nature of individualization defies the concept of consistent delivery of the intervention. This is a common challenge addressed in process evaluations of complex interventions with several active ingredients [2, 5, 29]. Carroll et al. argue that there is room for flexibility in delivery as long as the core components of an intervention are delivered [7]. This allotted flexibility is however not sufficiently specified. At what point will individualization of the intervention take on the form of a different intervention than the one described in the protocol? Any further assessment of implementation fidelity should focus on clarifying if there are limits to the possible individualization before it affects fidelity negatively. Another element of this challenge is the incongruity between research methodology in RCTs with the inherent demand for stringent and uniform delivery of interventions as opposed to the empirical evidence that supports individualization of interventions to patients with complex and heterogeneous medical conditions, such as stroke.

Though it is sensible to video record or observe a sample of intervention sessions to assess adherence and quality of delivery, we were not able to include this in the design of our process evaluation due to the financial and personnel constraints of the study. In an effort to ameliorate the lack of video recorded or observation material to evaluate the content of the sessions, the IP were instructed to keep log notes of each session. Unfortunately, the quality of the log notes was not sufficient for us to analyze them in any meaningful way. For future endeavors, the instructions on how to use the log notes would need to be clearly stated to ensure data of a higher quality. It may also be advisable to include the use of a structured checklist for IP to evaluate adherence to the protocol for each intervention session in the evaluation design. Audiotaping selected intervention sessions could also add valuable information with regard to content and quality of delivery.

In the planning stages of the process evaluation, the decision to use a conceptual framework to evaluate implementation fidelity was central. The conceptual framework for implementation fidelity was useful in raising consciousness of the complexity of implementation of the intervention. The conceptual framework also enabled a highly systematic analysis of adherence and potential moderators to implementation fidelity. One limitation was that, for the purpose of assessing implementation within the frame of an RCT, the framework does not account for assessing the control arm of the study. Although the control group is not a part of the intervention itself, the interpretation of results from the RCT will compare both arms of the trial. The assessment of issues concerning the control group would be of further use in forthcoming interpretation of RCT outcomes if more attention was paid to the control arm of the study. To address this lack of attention in the conceptual framework an assessment of how the data collection procedures and in-person assessment interviews may have influenced the outcomes reported by the control group participants was included in our process evaluation. This assessment will be presented in a forthcoming publication [30].

This is the first attempt to clarify the levels of fidelity in this intervention, and the reference values of low, medium, and high fidelity were set by the research team according to the guidelines of the protocol. The interpretation of the levels of fidelity is still in its pioneering stages. As discussed in Additional file 1, we made judgements regarding potential weighting of variables in the construction of the composite score of adherence. The feasibility studies did not give any indications to support weighting one variable over the others, thus, we constructed an equally weighted composite score. There may however have been other issues to consider in constructing this score. The choice to divide the fidelity measurement into three categories needs to be evaluated in further studies. It is possible that, given more experience in conducting the interventions and a better overview of individual adaptations and how these should be scored, the reference values would be set differently in future evaluations. We found the three-category solution to be advisable because it added distinction beyond the dichotomy of low vs. high fidelity, which we thought were too broad categories. However, looking at the results, the large proportion of the intervention trajectories that satisfied the criteria of high fidelity may be an indication that the reference values and/or the number of categories should be critically reviewed in future studies.

## Conclusions

Overall, the implementation fidelity in the RCT was high, 80% of the interventions were completed within the criteria of high fidelity. The results show that the core components of the intervention were delivered although the intervention trajectories were individualized. The biggest challenge was concluding the intervention within the time frame specified in the protocol. Although it is difficult to conclusively assess the importance of each of the moderating factors in relation to the other factors and to their influence on the adherence measures, five of the moderating factors identified in the conceptual framework [4, 7] appeared to be more prominent in this study. Participant responsiveness to the intervention, contextual factors, quality of delivery, and comprehensiveness of policy description were especially important moderators of content, frequency, and duration. Furthermore, recruitment and context had an important impact on the coverage in this study.

This evaluation of implementation fidelity and the discussion of what constitutes high fidelity implementation of this intervention are crucial in understanding the factors influencing the trial outcome. Moreover, this evaluation is imperative in planning the implementation of research findings in the primary care setting and in planning further studies.

## Additional files

Additional file 1: Implementation fidelity measurement. Description of scoring system to categorize values of variables within three fidelity categories; low, medium and high fidelity. Description of the construction of composite score outlining levels of fidelity. (PDF 553 kb)

Additional file 2: Overview of moderators, data sources and short excerpt of findings. This file provides an overview of the potential moderators, data sources used in the analysis, and a short summary of the findings to illustrate how the analysis of moderating factors was systematically conducted. (PDF 341 kb)

## Abbreviations

GHQ-28: General Health Questionnaire - 28
GSD: Guided self-determination
HCP: Health care professional
IP: Intervention personnel
NIHSS: The National Institutes of Health Stroke Scale
OT: Occupational therapist
RCT: Randomized controlled trial
RN: Registered nurse
SOC: Sense of Coherence
