# Supplementary material for: Implementation fidelity in a complex intervention promoting psychosocial well-being following stroke: an explanatory sequential mixed methods study
Source: BMC Med Res Methodol. 2019 Mar 15;19:59. doi: 10.1186/s12874-019-0694-z (PMC6419826; doi:10.1186/s12874-019-0694-z)
Supplement: Supplementary file 1 — Implementation fidelity measurement. Description of scoring system to categorize values of variables within three fidelity categories; low, medium and high fidelity. Description of the construction of composite score outlining levels of fidelity. (PDF 553 kb) [file 12874_2019_694_MOESM1_ESM.pdf]

Implementation fidelity measurement

We argue that the levels of fidelity can be categorized with three levels: Low fidelity, medium fidelity and high fidelity. Based on reference values from the study protocol, a scoring system was devised to categorize the values of each of the variables within one of three fidelity categories (Table 1).

Table 1: Levels of fidelity: variable reference values and fidelity scores

	Low Fidelity	Medium Fidelity	High Fidelity	Medium Fidelity	Low Fidelity
Content and coverage					
Number of sessions	1 – 3	4 – 5	6 - 8		
Frequency and duration					
Timely start[1]			0 - 60	61 - 75	≥76
Duration[2]	0 - 8	9 -12	13 - 21	22 - 25	≥ 26
Timely completion[1]	-150 - -50[3]	-49 - -31	- 30 – 0	1 – 15	≥16

A value within the category low fidelity was awarded 0 points, values within medium fidelity gives 1 point, and values within high fidelity gives 2 points.

The construction of a composite score outlining the levels of implementation fidelity was deemed appropriate. The results from the feasibility studies conducted as part of the development of the intervention gave no reason to differentiate or weight one or several variables over the others. An equally weighted additive composite score outlining levels of implementation fidelity was constructed adding the score from each of the four variables

[1] Measured in days
[2] Measured in weeks
[3] Negative numbers denotes days before the 6-month mark, and positive numbers denotes days after the 6-month mark.

measuring adherence. The composite variable was scored on a 9-point scale from minimum 0 to maximum 8. The composite variable values of 0-2 represents low fidelity, 3-5 represents medium fidelity, and 6-8 represents high fidelity (Table 2).

Table 2 Composite fidelity score

**Variable**	**Low fidelity**	**Medium fidelity**	**High fidelity**
Composite fidelity score	0-2	3-5	6-8
