# Supplementary material for: Implementation fidelity in a complex intervention promoting psychosocial well-being following stroke: an explanatory sequential mixed methods study
Source: BMC Med Res Methodol. 2019 Mar 15;19:59. doi: 10.1186/s12874-019-0694-z (PMC6419826; doi:10.1186/s12874-019-0694-z)
Supplement: Supplementary file 2 — Overview of moderators, data sources and short excerpt of findings. This file provides an overview of the potential moderators, data sources used in the analysis, and a short summary of the findings to illustrate how the analysis of moderating factors was systematically conducted. (PDF 341 kb) [file 12874_2019_694_MOESM2_ESM.pdf]

Additional file 2

Overview of moderators, data sources and short excerpt of findings

In the conceptual framework developed by Carroll et al. (2007) and modified by Hasson et al. (2010), factors that have the potential to influence implementation fidelity are referred to as moderators. Additional file 2 provides an overview of the potential moderators, data sources used in the analysis, and a short summary of the findings to illustrate how the analysis of moderating factors was systematically conducted.

**Moderator**		**Description**	**Data sources**	**Findings**
Participant responsiveness	Patients' responsiveness	Refers to how well patients who are receiving the intervention respond to or are engaged by an intervention. Includes judgement about relevance and outcomes.	Fourteen individual qualitative interviews with patients from the intervention arm of the RCT.<br><br>Five focus group interviews, including 17 healthcare professionals delivering the intervention (IP).<br><br>Attrition records.	Motivation for participation varied: own needs for help; contribution to research, and altruistic motives (wanted to help others in the same situation). Nearly all the interviewees confirmed the relevance of a psychosocial intervention after stroke. All, except two, of the participants expressed benefits in terms of being listened to; receiving response and advice from dedicated health professionals; gaining knowledge and information about stroke; experiencing feelings of safety and security in knowing that they were receiving follow-up. Two did not benefit from the intervention as they felt they had fully recovered before the intervention started.
	Intervention personnel's responsiveness	Refers to how healthcare professionals who delivered the intervention were engaged by the intervention. Includes judgement about relevance and outcomes.	Five focus group interviews, including 17 healthcare professionals delivering the intervention (IP).<br><br>Fourteen individual qualitative interviews with patients from the intervention arm of the RCT.	The healthcare professionals delivering the intervention were all highly motivated for conducting the interventions. They all expressed the need for programs targeting psychosocial needs in stroke rehabilitation. Organising the meetings and making appointments with the patients were performed differently; some scheduled all the 8 sessions in line with the structure in the guiding manual at the beginning of the intervention trajectory, while others made appointments from session to session.

Additional file 2

Comprehensiveness of policy description	Intervention complexity and to which degree the intervention is sufficiently and clearly described.	Five focus group interviews, including 17 healthcare professionals delivering the intervention (IP).	Development of the intervention and its theoretical and empirical foundation was developed in line with the UK MRC guidance on development and evaluation of complex interventions. A detailed manual describing the content and the suggested structure of each of the sessions in the intervention trajectory was supplied. The IP were certified through a 3-day training program consisting of lectures, practical training exercises, group reflection and discussions, and individual reading of specific literature. Development of the intervention and its theoretical and empirical foundation was part of the lectures. The IP described the manual as a useful tool that they used systematically in their intervention delivery.
Strategies to facilitate implementation	Supporting strategies, which include standardised written procedures, training programs and guidelines to optimise a delivery to be as uniform as possible.	Trial coordinators' records.<br><br>Five focus group interviews, including 17 healthcare professionals delivering the intervention (IP).	The manual describing the content and structure of the intervention was an important part of the strategy to facilitate implementation. Additionally, the IP were offered supervision individually and in groups during the intervention delivery to facilitate uniform delivery. The written procedures, training program, and supervision during the study period were evaluated as informative and adequate.
Quality of delivery	Concerns whether an intervention was delivered in a way appropriate to achieving what was intended.	Five focus group interviews, including 17 healthcare professionals delivering the intervention (IP).<br><br>Fourteen individual qualitative interviews with patients from the intervention arm of the RCT.	IP had, on average, 9.8 years clinical experience working with stroke patients. They were concerned about delivering the core components of the intervention. However, the number of interventions conducted by each IP varied from 1 to 33. Some of the IP felt they had too few interventions to become sufficiently confident with the theoretical and methodological foundation of the intervention and they wished for more experience through additional interventions.<br>The complexity of the patients' needs challenged the IP role in some trajectories, and the patients' responses to the themes and worksheets varied, resulting in heterogeneous trajectories. The number (27) of IP delivering the intervention may challenge the ideal of a uniform delivery, but we lack sufficient data to evaluate this in detail. However, the patients appreciated the relationship and dialogues with the IP and characterized the IP as knowledgeable, empathetic and attentive.

Additional file 2

Recruitment	Recruitment procedures and consistency of recruitment procedures among eligible patients, and reasons for non-participation.	Enrolment records.<br><br>Attrition records.<br><br>Trial coordinators' records	Recruitment was slow and not sufficiently consistent. Enrolment records indicate that the number of patients screened for eligibility were insufficient compared to the actual number of patients with stroke in each of the participating centres. Other competing studies targeting the same population was a substantial concern in a few of the hospitals. Recruitment were not a prioritized task among several duties and activities in a hospital. Staff turnover, shift work and vacation hampered the systematic screening for eligible patients.
Context	Surrounding social systems, structures and cultures of organization and concurrent events	Five focus group interviews, including 17 healthcare professionals delivering the intervention (IP).<br><br>Trial coordinators' records.	In addition to the intervention, the patients received regular rehabilitation services in the municipality and in secondary health care institutions, resulting in busy schedules for the patients and sometimes problems with finding time that matched the IP schedules. Vacation and sick leave also came in conflict with the proposed frequency and duration in some trajectories. The IP delivering the intervention were voluntary participants who delivered the intervention as an addition to their ordinary work.
